# Systemic therapy of metastatic breast cancer: a plea for confirmation of subtypes by genomic assays

**DOI:** 10.1007/s00432-022-03989-0

**Published:** 2022-04-12

**Authors:** Diana Lüftner

**Affiliations:** Department of Hematology, Oncology and Tumor Immunology, Charité Campus Benjamin Franklin, University Medicine Berlin, Hindenburgdamm 30, 12200 Berlin, Germany

Any targeted therapy can only be as good as the underlying diagnostics. Hormonal treatment of endocrine sensitive metastatic breast cancer was the first targeted therapy in oncology. We rely on immunohistochemistry (IHC) of the hormone receptors (HR) and re-consider a biopsy of a metastatic lesion should the protein expression have shifted since primary disease. Cutoffs of positivity have often been changed based on technical approaches and academic definitions, but clinically any HR receptor expression > 10% of tumor cells is considered positive and warrants antihormonal treatment.

Recently, a genomic assay and overall survival analysis of the MONALEESA2/3/7 studies (intention to treat—ITT population) was presented using a modified PAM50-based genomic assay to define the molecular subtypes of these patients who were treated with a combination of the CDK4/6 inhibitor ribociclib plus endocrine therapy (ET: aromatase inhibitor or fulvestrant) for first—or second-line treatment of metastatic breast cancer (Carey et al. 2022). Multivariate models were adjusted for known clinical factors. A total of 997 samples of this HR positive population by IHC (biomarker population) yielded 82.3% of luminal A or B tumors, 14.7% of HER2 enriched tumors and 3.0% of basal-like subtypes.

While the survival benefit of the ITT population and the biomarker population yielded the same overall survival benefit adding ribociclib to ET, the intrinsic subtype was prognostic for OS with adjusted hazard ratios (HR) in multivariate analysis of HR = 0.77 for luminal A subtypes, HR = 0.63 for luminal B subtypes, HR = 0.53 for HER2-enriched and HR = 2.71 for basal-like tumors.

It is clear that the outcome of basal-like breast cancer is always poor, but we have to keep in mind that these patients did not receive any adequate systemic therapy with ET plus ribociclib, and we must assume that this was true for more than one line of „falsely chosen “ETs. In addition, potential biopsies—also for later confirmation of the HR status and subsequent endocrine treatments—may have given another biologically misleading IHC result. The situation is marginally better for the HER2-enriched subtype: CDK4/6 inhibitors are known to be active in HER2 positive breast cancer, and moreover, the HER2 enriched subtype also includes triple-positive tumors in which ET is effective. Nevertheless, it must be assumed that patients with HER2 enriched tumors would have had benefited from the addition of anti-HER2-targeted therapies, before all trastuzumab, in this early line setting of metastatic disease.

We accept undertreating approximately 18% of these formally HR + metastatic breast cancer patients in clinical trials as well as in the real-time setting and neglect a baseline selection mistake into systemic therapies without a confirmation of the IHC results by a genomic assay. In addition, with all the current evidence of similar results in literature, it seems ethically impossible to do a randomized clinical trial to clarify the situation (Jørgensen et al. 2021; Cejalvo et al. 2017; Schettini et al. 2021). Example given: in case of discordant results between positive HR status by IHC and molecular genomic assay result, similar to the TAILOR-X or MINDACT trials in early breast cancer, it would not be ethical to randomize basal-like patients with positive IHC for HR to endocrine therapy or not. No patient or physician should be willing to participate in such an experiment.

The solution to the problem is hard to swallow. We must bring genomic assays for confirmation of the molecular subtype into clinical routine to avoid undertreatment of a substantial subset of patients with metastatic breast cancer. We can only get data from prospective registries to assess the undertreatment associated with pure IHC selection of metastatic breast cancer patients in comparison with genomic profiling. We have to keep in mind: any targeted therapy can only be as good as the best underlying diagnostics.
